# Factors associated with incomplete surgical excision of non-melanoma skin cancers: a single-center retrospective cross-sectional study

**DOI:** 10.1097/MS9.0000000000004954

**Published:** 2026-06-09

**Authors:** Yeganeh Pakbaz, Sepideh Eydivandi, Golnoosh Seirafianpour, Farnoosh Seirafianpour, Kimia Ghanavati, Azadeh Goodarzi

**Affiliations:** a Department of Dermatology, Rasool Akram Medical Complex Clinical Research Development Center (RCRDC), School of Medicine, Iran University of Medical Sciences, Tehran, Iran; b Gastrointestinal and Liver Diseases Research Center, School of Medicine, Iran University of Medical Sciences, Tehran, Iran; c Department of Biotechnology, Islamic Azad University, Tehran Central Branch, Tehran, Iran; d Razi Drug Research Center, Iran University of Medical Sciences, Tehran, Iran; e Otorhinolaryngology Research Center, Department of Otolaryngology and Head and Neck Surgery, School of Medicine, Guilan University of Medical Sciences, Rasht, Iran

**Keywords:** basal cell carcinoma, basosquamous carcinoma, cross-sectional study, Mohs micrographic surgery, non-melanoma skin cancer, squamous cell carcinoma

## Abstract

**Background:**

Non-melanoma skin cancers (NMSCs), including basal cell carcinoma (BCC) and squamous cell carcinoma (SCC), are among the most common malignancies worldwide. Complete surgical excision is crucial for minimizing recurrence and improving outcomes. However, challenges remain, particularly when margins are unclear, which leads to higher clinical and economic burdens.

**Methods:**

This retrospective cross-sectional study analyzed factors associated with incomplete excision rates (IERs) in 448 NMSC lesions excised from 413 patients between 2017 and 2022. Patient demographics, lesion characteristics, anatomical site, practitioner specialty, and histopathological data were assessed. Statistical analyses, including chi-square and *t*-tests, were performed using SPSS version 22, with *P* < 0.05 considered significant.

**Results:**

The overall IER was 29.0%. Younger patients (mean age: 64.2 years) had a significantly higher IER than older patients (*P* = 0.026). In BCC cases, smaller lesions (≤10 mm) had a significantly higher IER (31.5%, *P* = 0.043), and excisions performed by dermatologists had the highest IER (31.9%, *P* = 0.045). General surgery had the lowest IER (8.3%). Gender was not significantly associated with IER (*P* = 0.968). Most lesions (92.4%) were located on the head and neck, though the anatomical site did not significantly affect IER.

**Conclusion:**

Incomplete excision remains a significant challenge in the surgical management of NMSC, particularly in younger patients, small BCC lesions, and cases treated by dermatologists. These findings highlight the need for a more meticulous surgical approach, particularly in high-risk cases. Optimizing surgical margins, refining excision techniques, and improving training protocols may help reduce IER and enhance patient outcomes.

## Introduction

Non-melanoma skin cancers (NMSCs), also referred to as keratinocyte cancers, are among the most prevalent human malignancies, with their incidence continuing to rise globally[1]. NMSCs encompass all malignant skin tumors that are not classified as melanoma. The two most common types, basal cell carcinoma (BCC) and squamous cell carcinoma (SCC), collectively account for approximately 99% of all NMSCs^[^2,3^]^. In rare cases, hybrid lesions with features of both BCC and SCC, known as basosquamous carcinomas (BSC), are observed^[^4,5^]^. Less frequent subtypes include sebaceous carcinoma, Merkel cell carcinoma, apocrine adenocarcinoma, and other rare skin tumors^[^6,7^]^. Although BCC is highly treatable, its tendency for recurrence poses a notable clinical challenge[8].

HIGHLIGHTS

- Younger patients had significantly higher incomplete excision rates (IERs).
- Lesion size ≤10 mm had the highest IER, especially in basal cell carcinoma.
- Dermatologists had the highest IER (32.0%) among specialties.
- The head and neck were the most affected anatomical sites (92.4%).
- No significant IER differences by sex were observed.
- Optimizing surgical margins and improving practitioner training may help reduce IERs in non-melanoma skin cancer cases.

Achieving clear surgical margins is crucial in reducing the recurrence rates of BCC. Studies have demonstrated that when margins are clear, the recurrence rate for BCC is approximately 1–3% for primary tumors. In contrast, tumors with involved margins exhibit significantly higher recurrence rates, ranging from 26% to 38%^[^9–11^]^. These findings underscore the importance of complete tumor excision to minimize the risk of recurrence. Although fewer data are available for other types of NMSC, complete excision is similarly critical due to the lesion’s metastatic potential. For instance, recurrence rates for SCC range from 5% to 47%, depending on histological and clinical factors[12].

Despite the availability of various treatment options, surgical approaches remain the most effective treatment for NMSC. Common surgical techniques include curettage and electrodesiccation (C&E), Mohs micrographic surgery (MMS), and standard surgical excision (SE). The primary objectives of surgical intervention are to ensure complete tumor removal, prevent recurrence, minimize functional impairment, and achieve the best possible cosmetic outcomes^[^13–15^]^. When selecting excision margins for surgical approaches, it is crucial to balance complete tumor clearance with the need to avoid removing excessive healthy tissue. The size of the excision margin is closely linked to the risk of recurrence and must be carefully considered[16].

Incomplete excisions may require additional surgeries or closer surveillance, placing a burden on both patients and healthcare systems, while also increasing the morbidity and cost of skin cancer management^[^1,15^]^. Defining safe surgical margins and understanding their impact on prognosis and recurrence are essential for developing evidence-based strategies to reduce incomplete excisions and improve patient outcomes^[^17,18^]^. Factors contributing to incomplete excisions include tumor location, multifocality, operator expertise, and histopathological evaluation errors. Addressing these factors is essential to improving surgical outcomes and preventing recurrence^[^15,19^]^.

This study aims to evaluate the epidemiological, clinical, histopathological, and other relevant factors associated with incomplete surgical excisions of NMSC lesions. The ultimate goal is to develop strategies to reduce incomplete resections, thereby improving patient outcomes and optimizing healthcare resource utilization.

This cross-sectional study has been reported in line with the STROCSS guidelines[20].

## Materials and methods

### Study design

This retrospective cross-sectional study was conducted on patients treated for NMSCs at Rasool Akram Medical Complex, Tehran, Iran, a tertiary teaching hospital, over 5 years, from March 2017 to February 2022.

### Patient selection

Medical records of patients diagnosed with NMSCs were retrieved from the hospital’s electronic database. Eligible patients had a confirmed histopathological diagnosis of BCC, SCC, or BSC. Incomplete excision was defined as the presence of tumor cells at the inked surgical margin on histopathological examination, as reported by two independent dermatopathologists according to the College of American Pathologists (CAP) Cancer Protocols[21]. Inclusion criteria comprised patients undergoing full-thickness excision. Cases with missing histopathological reports or insufficient clinical documentation were excluded from the study. Patients lost to follow-up, with unclear excision status or treatments other than full-thickness excision (e.g., punch biopsy, frozen section, shave excision, incisional biopsy, cryotherapy, or C&E), were also excluded to prevent bias. Statistical handling of missing data involved sensitivity analysis to assess the impact of exclusions on study outcomes. Patients were followed up regularly for a minimum of 2 years, every 6 months.

Detailed demographic and clinical data of 413 patients were collected, including patient age, sex, anatomical site, lesion size, and the medical specialty of the clinician performing the excision. Lesion dimensions were stratified into three predefined categories based on their measured size: ≤10, 10–20, and ≥20 mm.

### Immunohistochemical analysis

Histological specimens were stained with hematoxylin and eosin (H&E) and independently examined by two dermatopathologists. Immunohistochemical analysis was performed when further differentiation between tumor subtypes was required. Ber-EP4 was used to distinguish BCC from other skin conditions, such as SCC and BSC. CK5/6 and p63 were applied to confirm SCC and differentiate it from other epithelial tumors. Additionally, epithelial membrane antigen (EMA) was used to distinguish SCC from BCC, ensuring precise classification and diagnosis.

### Ethical considerations

The data were anonymized using encrypted identifiers. All procedures were conducted in accordance with the Declaration of Helsinki.

### Statistical analysis

Data analysis was conducted using SPSS version 22. Quantitative variables, such as age, were reported as mean ± standard deviation (SD), while categorical variables, including sex, lesion site, and size, were expressed as frequencies and percentages. Comparative analysis was performed using the chi-square test or Fisher’s exact test for categorical variables and the t-test for continuous variables, depending on data distribution. Subgroup analyses were conducted based on clinically relevant variables identified in previous studies, including age, lesion size, tumor type, anatomical site, and practitioner specialty. Incomplete excision rates (IERs) were calculated for each subgroup, and associations were examined using univariate statistical methods. Statistical significance was defined as a *P*-value < 0.05.

## Results

This study analyzed 448 NMSC lesions excised from 413 patients at Rasool Akram Medical Complex Hospital. The cohort included 308 BCC, 130 SCC, and 10 BSC lesions, treated in 280, 123, and 10 patients, respectively. The overall IER for all NMSC lesions was 29.0% (130 of 448). The mean age of all patients was 66.66 years (median: 68, range: 11–94 years). Patients with incomplete excisions were significantly younger (mean age: 64.19 years; *P* = 0.026) than those with complete excisions (mean age: 67.67 years). Males constituted 66.3% of the study population, with a male-to-female ratio of 1.97:1. There was no significant difference in IER between sexes, with rates of 29.0% in men and 29.1% in women (Table 1).

**Table 1:** Patient demographics and incomplete excision rates by tumor type.

	BCC			SCC			BSC			All NMSC		
	Incomplete	Total	*P*-Value	Incomplete	Total	*P*-Value	Incomplete	Total	*P*-Value	Incomplete	Total	*P*-Value
N	81	308		46	130		3	10		130 (29.0)	448	
Age (years)												
Mean	63.41 ± 16.39	66.45 ± 13.20	0.039^a^	65.15 ± 15.34	66.98 ± 14.19	0.280	68 ± 6.43	68.80 ± 7.95	0.655	64.19 ± 15.844	66.66 ± 13.385	0.026^a^
Median	66	68		67.50	68		68	69.50		66.00	68.00	
Range	20–71	11–94		22–91	14–93		66–78	53–78		20–91	11–94	
Age group (years)	N (%)	N		N (%)	N		N (%)	N		N (%)	N	
≤ 50	13 (37.1)	35	0.122^a^	7 (50.0)	14	0.247^b^	0 (0.0)	0	-	20 (40.8)	49	0.054^a^
50–75	48 (23.8)	202	0.163^a^	27 (32.5)	83	0.366^a^	2 (28.6)	7	1^b^	77 (26.4)	292	0.091^a^
≥ 75	20 (28.2)	71	0.683^a^	12 (36.4)	33	0.892^a^	1 (33.3)	3	1^b^	33 (30.8)	107	0.636^a^
Sex												
Male	51 (25.6)	199	0.718^a^	33 (35.5)	93	0.970^a^	2 (40.0)	5	1^b^	86 (29.0)	297	0.968^a^
Female	30 (27.5)	109		13 (35.1)	37		1 (20.0)	5		44 (29.1)	151	

BCC, basal cell carcinoma; BSC, basosquamous carcinoma; NMSC, non-melanoma skin cancer; SCC, squamous cell carcinoma.

^a^ Chi-squared test.

^b^ Fisher’s exact test.

## Incomplete excision by lesion size

The IER in all NMSC lesions varied according to lesion size, with rates of 32.0% for lesions ≤10 mm, 30.9% for lesions 10–20 mm, and 23.6% for lesions ≥20 mm. These differences were not statistically significant (Table 2).

**Table 2:** Incomplete excision rates by lesion size, anatomical site, and practitioner specialty.

	BCC			SCC			BSC			All NMSC		
	Incomplete	Total	P-Value	Incomplete	Total	P-Value	Incomplete	Total	P-Value	Incomplete	Total	P-Value
	N (%)	N		N (%)	N		N (%)	N		N (%)	N	
Size of lesion (mm)												
≤ 10	47 (31.5)	149	0.043^a^	15 (33.3)	45	0.722^a^	0	0	-	62 (32.0)	194	0.231^a^
10–20	18 (23.7)	76	0.551^a^	15 (48.4)	31	0.083^a^	1 (33.3)	3	1^b^	34 (30.9)	110	0.615^a^
≥ 20	16 (19.3)	83	0.089^a^	16 (29.6)	54	0.247^a^	2 (28.6)	7	1^b^	34 (23.6)	144	0.083^a^
Anatomical site												
Head & Neck	76 (25.7)	296	0.312^b^	40 (37.0)	108	0.383^a^	3 (30.0)	10	-	119 (28.7)	414	0.656^a^
Ears	12 (37.5)	32	0.105^a^	8 (34.8)	23	0.801^a^	1 (50.0)	2	1^b^	21 (36.8)	57	0.146^a^
Eyes	20 (24.4)	82	0.754^a^	5 (38.5)	13	1^b^	1 (25.0)	4	1^b^	26 (26.3)	99	0.532^a^
Nose	12 (23.1)	52	0.637^a^	2 (28.6)	7	1^b^	1 (50.0)	2	1^b^	15 (24.6)	61	0.438^a^
Forehead	5 (21.7)	23	0.653^a^	3 (37.5)	8	1^b^	-	-	-	8 (25.8)	31	0.707^a^
Scalp	19 (26.8)	71	0.810^a^	8 (32.0)	25	0.552^a^	0 (0.0)	2	1^b^	27 (27.6)	98	0.765^a^
Cheeks, lips, chin	7 (23.3)	30	0.757^a^	8 (38.1)	21	0.911^a^	-	-	-	15 (29.4)	51	0.910^a^
Neck	1 (16.7)	6	1^b^	6 (54.5)	11	0.323^b^	-	-	-	7 (41.2)	17	0.276^b^
Trunk	4 (57.1)	7	0.081^b^	0 (0.0)	3	0.552^b^	-	-	-	4 (40.0)	10	0.485^b^
Lower extremities	0 (0.0)	1	1^b^	2 (40.0)	5	1^b^	-	-	-	2 (33.3)	6	1^b^
Upper extremities	1 (25.0)	4	0.569^b^	4 (28.6)	14	0.769^b^	-	-	-	5 (27.8)	18	0.906^a^
Ward												
Dermatology	44 (31.9)	138	0.045^a^	18 (33.3)	54	0.680^a^	0 (0.0)	2	1^b^	62 (32.0)	194	0.231^a^
ENT	18 (22.2)	81	0.332^a^	24 (38.7)	62	0.449^a^	2 (40.0)	5	1^b^	44 (29.7)	148	0.816^a^
Ophthalmology	17 (22.1)	77	0.331^a^	3 (37.5)	8	1^b^	1 (33.3)	3	1^b^	21 (23.9)	88	0.235^a^
Surgery	1 (12.5)	8	0.686^b^	0 (0.0)	4	0.296^b^	-	-	-	1 (8.3)	12	0.193^b^
Others	1 (25.0)	4	1^b^	1 (50.0)	2	1^b^	-	-	-	2 (33.3)	6	1^b^

BCC, basal cell carcinoma; BSC, basosquamous carcinoma; NMSC, non-melanoma skin cancer; SCC, squamous cell carcinoma.

^a^ Chi-squared test.

^b^ Fisher’s exact test.

## Anatomical distribution

The majority of NMSC lesions (92.4%) were located on the head and neck, with an IER of 28.7% for lesions in this region. Other anatomical sites included the trunk (IER: 40.0%), the upper extremities (IER: 33.3%), and the lower extremities (IER: 27.8%). Lesion location did not significantly affect the IER (Table 2).

## Practitioner specialty and ward distribution

IER varied across specialties and wards, with dermatology exhibiting an IER of 32.0%, otolaryngology at 29.7%, ophthalmology at 23.9%, general surgery at 8.3%, and other wards at 33.3% for all NMSC lesions. However, these differences were not statistically significant (Table 2).

**Figure 1.**
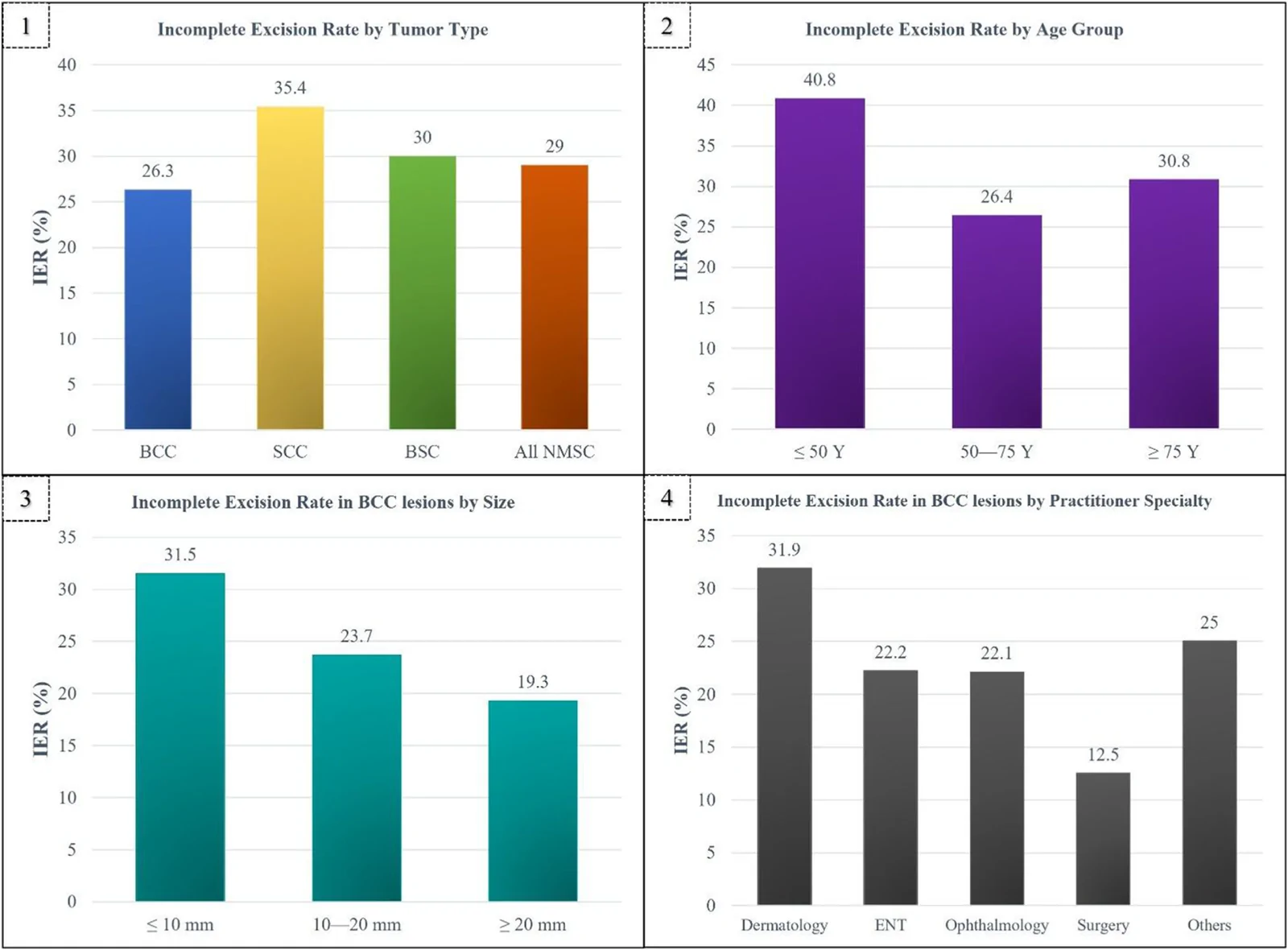
Incomplete excision rates by tumor type, age group, lesion size, and practitioner specialty. (1) Incomplete excision rate by tumor type, (2) by age group, (3) in BCC lesions by size, and (4) in BCC lesions by practitioner specialty.

Results are further detailed by lesion type, with an analysis of age, gender, lesion size, anatomical site, and practitioner specialty contributing to the IERs (Fig. 1).

## Lesion type-specific findings

### Basal cell carcinoma

#### Patient demographics

The mean age of patients with BCC lesions was 66.45 years (range: 11–94 years). Patients with incomplete excision were significantly younger, with a mean age of 63.41 years compared to 67.54 years for those with complete excision (*P* = 0.039). Of the 308 BCC lesions, 64.6% were in men and 35.4% in women, yielding a male-to-female ratio of 1.82:1. The IER was 25.6% for men and 27.5% for women, with no significant gender difference (Table 1).

#### Lesion size and anatomical site

The IER was 31.5% for lesions ≤10 mm, 23.7% for lesions 10–20 mm, and 19.3% for lesions ≥20 mm. A statistically significant difference was observed only for lesions ≤10 mm (*P* = 0.043). Anatomical analysis revealed that 96.4% of BCC lesions were located on the head and neck, 2.3% on the trunk, 0.3% on the lower extremities, and 1.0% on the upper extremities. The IER was 25.7% for lesions on the head and neck, 57.1% on the trunk, 25.0% on the upper extremities, and 0% on the lower extremities, with no significant differences based on anatomical site (Table 2).

#### Practitioner specialty

The IER varied by specialty, with rates of 31.9% in the dermatology ward, 22.2% in otolaryngology, 22.1% in ophthalmology, 12.5% in surgery, and 25.0% in other wards. A statistically significant difference was observed only in the dermatology ward (*P* = 0.045) (Table 2).

### Squamous cell carcinoma

#### Patient demographics

The mean age of patients with SCC lesions was 66.98 years (range: 14–93 years). Patients with incomplete excision had a mean age of 65.15 years, compared to 67.98 years for those with complete excision, though this difference was not statistically significant. Of the 130 SCC lesions, 71.5% were in men and 28.5% in women, with a male-to-female ratio of 2.51:1. The IER was 35.5% for men and 35.1% for women, with no significant gender difference (Table 1).

#### Lesion size and anatomical site

The IER was 33.3% for lesions ≤10 mm, 48.4% for lesions 10–20 mm, and 29.6% for lesions ≥20 mm, with no statistically significant differences across size groups. Anatomically, 83.1% of SCC lesions were located on the head and neck, 2.3% on the trunk, 3.8% on the lower extremities, and 10.8% on the upper extremities. The IER was 37.0% for head and neck lesions, 0% for trunk lesions, 40.0% for lower extremity lesions, and 28.6% for upper extremity lesions, with no significant differences based on anatomical location (Table 2).

#### Practitioner specialty

The IER for SCC varied across specialties, with rates of 33.3% in dermatology, 38.7% in otolaryngology, 37.5% in ophthalmology, 0% in surgery, and 50.0% in other wards. However, these differences were not statistically significant (Table 2).

### Basosquamous Carcinoma (BSC)

#### Patient demographics

The mean age of patients with BSC lesions was 68.80 years (range: 53–78 years). There was no significant age difference between the incomplete and complete groups. Among the 10 BSC lesions, the gender distribution was equal, with a male-to-female ratio of 1:1. The IER was 40.0% in men and 20.0% in women, though this difference was not statistically significant (Table 1).

#### Lesion size and anatomical site

The IER for BSC lesions was 0% in lesions ≤10 mm, 33.3% in lesions 10–20 mm, and 28.6% in lesions ≥20 mm, with no significant differences observed across the size groups. All BSC lesions were located in the head and neck region. While the IER varied across specific anatomical sites within this region, the differences were not statistically significant (Table 2).

## Practitioner specialty

The IER for BSC lesions was 0% in the dermatology department, 40% in otolaryngology, and 33.3% in ophthalmology, with no significant differences by specialty. No BSC lesions were treated in the surgical or other departments (Table 2).

## Discussion

This study provides a comprehensive analysis of factors associated with incomplete surgical excision of NMSCs, highlighting the importance of patient demographics, lesion characteristics, anatomical sites, and practitioner specialties in determining excision outcomes. These results underscore the persistent challenges in achieving a clear margin and highlight the need for tailored strategies to optimize surgical outcomes.

The IER of NMSC lesions in our study aligns closely with findings in previous literature[22]. Dalal *et al*[23] reported a rate of 10.8% for incomplete excisions, whereas van Rijsingen *et al*[24] observed a higher rate of 35% (65.4% in the face and neck region). The latter is consistent with our results. In our analysis, among the NMSC subtypes, the IER was 26.3% for BCC and 35.4% for SCC lesions, both exceeding rates reported in earlier studies. Recent research reported an IER of 11.0% for BCC and 9.4% for SCC[22]. These discrepancies highlight potential regional, procedural, or institutional differences in surgical practices and outcomes. These findings are clinically significant, particularly given the distinct biological behaviors and recurrence risks associated with these subtypes. For example, SCC’s metastatic potential warrants a more stringent surgical approach compared to BCC, which typically has a lower risk of metastasis but a high recurrence rate in incompletely excised cases.

These findings highlight the complexities of achieving complete excision in different age groups. Younger patients may present unique challenges, which may reflect variations in tumor biology, concerns regarding cosmetic outcomes, or issues related to patient compliance. However, contrary to this, existing studies indicate that older patients, particularly those with BCC, face a significantly higher risk of incomplete excision – a trend not observed in SCC lesions^[^25,26^]^. This variation suggests that age-related factors may influence surgical outcomes differently depending on the type of NMSC, warranting further investigation.

Our findings suggest that lesion size did not significantly influence the IER for overall NMSC, aligning with recent studies^[^27,28^]^. This contrasts with Bhatti *et al*[29], who reported a strong correlation between larger NMSC lesions and higher rates of positive residual margins. Interestingly, our study found a significant association between smaller BCC lesions (≤10 mm) and increased IER, suggesting that these lesions, often considered less complex, may receive less meticulous attention during excision. This observation is consistent with Codazzi *et al*, who identified an increased risk of incomplete excision in BCC lesions measuring ≤5 mm[30]. These findings emphasize the importance of avoiding underestimation of small lesions and ensuring adequate surgical margins of 3–5 mm during resection[9]. On the other hand, Kjerkegaard *et al* identified larger BCC lesions (≥ 15 mm) as a risk factor for incomplete resection[31]. For SCC lesions, our study did not find a significant correlation between lesion size and incomplete excision. This result aligns with most studies^[^31–34^]^ but contrasts with some others^[^35–39^]^, which suggest that larger lesion sizes (>20 mm) are associated with higher rates of incomplete excision.

Our findings are consistent with those of Kjerkegaard *et al*[31] who showed no significant correlation between anatomical site and IER. However, the head and neck regions were the most frequent sites of incomplete excisions, likely due to the predilection of NMSCs for sun-exposed areas and the challenges associated with surgical closure^[^32,40^]^. The high prevalence of IERs in these functionally and cosmetically sensitive regions reflects the difficulties in achieving adequate surgical margins, particularly in small or anatomically complex areas. This observation aligns with previous studies, which have reported the ears and the central facial zones as the most common anatomical sites for incomplete excisions^[^25,27,41,42^]^.

The variation in IER across medical specialties is a critical finding. Although multiple studies have established that general practitioners (GPs) have the highest rate of incomplete excisions in NMSC lesions, with GPs being four times more likely to perform incomplete excisions for both BCC and SCC compared to dermatologists^[^22,39^]^, there remains controversy regarding the differences between other specialties[43]. Our study specifically compared specialists and found that BCC excisions performed by dermatologists in the dermatology ward were associated with higher IERs. These differences likely reflect variations in training, experience, and case complexity. Dermatologists frequently operate on cosmetically sensitive areas where conservative margins are prioritized, which may inadvertently increase the likelihood of incomplete excision. In contrast, previous studies by Nolan *et al*[22] and Svensson[39] have reported lower IERs in dermatology.

Our study offers a direct comparison of IERs among medical specialists, providing valuable insight into this understudied area. However, several limitations should be acknowledged. The retrospective design and single-center setting may limit the generalizability of our findings. Additionally, variability in histopathological reporting and follow-up duration could introduce bias. The absence of complete data and an insufficient number of outcome events precluded the use of multivariable logistic regression analysis, and the results, therefore, rely on univariate associations that should be interpreted cautiously. Moreover, margin status was documented only as involved or uninvolved, and more detailed subclassification (lateral vs deep or focal vs. extensive involvement) was not consistently available for analysis. Future multicenter, prospective studies with standardized protocols are needed to validate our results and investigate innovative interventions. Furthermore, surgical technique variability may have influenced IER because differences in how surgeons define and achieve margins were not analyzed. Notably, all patients in our study were treated by specialists, with no excisions performed by GPs. As a result, we were unable to compare IERs between specialists and GPs, which may limit direct comparisons with studies that included GP-performed excisions.

Surgeon- and context-related factors may also have influenced our findings. In a retrospective dataset, “practitioner specialty” likely captures a complex mix of surgeon-level variability (experience, case volume, and comfort with oncologic margins), technique-level differences (orientation/handling of specimens, use of dermoscopy or loupe magnification, depth and shape of excision, and approach to margin selection in high-risk subclinical spread), and contextual case complexity (referral patterns, recurrent or ill-defined tumors, tumors near functionally/cosmetically critical structures, and patient-specific constraints such as anticoagulation or limited reconstructive options). These factors may partly explain inter-specialty differences in IERs and could not be fully disentangled in the current design. Future prospective multicenter studies should incorporate surgeon identifiers, standardized excision protocols, and detailed case-mix adjustment (including tumor risk features and reconstruction complexity) to better quantify the independent contribution of surgeon variability and technique to incomplete excision.

The observed associations suggest several practical strategies to reduce incomplete excisions. First, small BCCs should not be considered “low-effort” lesions; careful clinical delineation (e.g., dermoscopy when available) and adherence to guideline-consistent margins may be particularly important for lesions ≤10 mm. Second, in anatomically complex or cosmetically sensitive head-and-neck areas, clinicians may consider margin-control approaches (e.g., Mohs micrographic surgery where available, staged excision, or intraoperative margin assessment in selected cases) when tumors are ill-defined, high-risk, or when tissue-sparing decisions may increase the risk of positive margins. Third, our findings support cross-specialty standardization, including shared local protocols for margin selection, specimen orientation, and pathology communication, alongside targeted skills training (especially for units managing higher-risk facial tumors). Together, these steps may decrease the re-excision burden, reduce recurrence risk, and improve patient-centered cosmetic and functional outcomes.

Collectively, our findings underscore the influence of patient demographics, lesion characteristics, and surgical approach on IERs. Younger patients may have more aggressive tumor biology and are often treated with conservative margins due to cosmetic concerns, particularly in facial lesions, increasing the risk of residual tumor. Clinicians may also underestimate malignancy in younger patients, leading to inadequate excision. Similarly, smaller lesions pose challenges in delineating tumor boundaries, and their subclinical extensions may be overlooked, necessitating wider margins. Histopathological assessment of small specimens can further complicate margin evaluation. Notably, dermatology had the highest IER for BCC, likely due to the prioritization of cosmetic outcomes.

Given the significant risk of recurrence associated with incompletely excised NMSCs, a proactive approach is essential to minimize IER. The integration of advanced preoperative imaging modalities, such as confocal microscopy, alongside intraoperative frozen section analysis and specialized surgical training, particularly in MMS, may significantly enhance surgical precision^[^44–46^]^. Additionally, optimizing intraoperative visualization through adequate lighting and magnification further contributes to the accuracy of tumor excision, thereby improving patient outcomes. These approaches are particularly valuable in high-risk cases and in anatomically or cosmetically sensitive areas where margin clearance is challenging[46]. Enhancing surgical expertise across specialties, particularly in dermatology and otolaryngology, may help reduce incomplete excisions[47]. Moreover, younger patients and those with smaller lesions may benefit from more individualized surgical planning to account for their specific risk factors for incomplete excisions[22]. Furthermore, improving patient education regarding the importance of achieving clear surgical margins could enhance adherence to recommended treatment protocols, ultimately supporting better long-term management of NMSCs.

In conclusion, this study identified lesion size (≤10 mm) and excisions performed by dermatologists as significant risk factors for incomplete excision in BCC lesions. Additionally, younger patient age was found to be a notable risk factor for incomplete excision across NMSC lesions. These findings underscore the need for careful evaluation of margins, particularly for high-risk lesions. Factors such as younger age (<50 years) and head and neck localization may contribute to higher IERs but require further investigation to establish definitive associations.

## Data Availability

The datasets used and/or analyzed during the current study are available from the corresponding author upon reasonable request.

## Research registration unique identifying number (UIN)

IR.IUMS.FMD.REC.1400.166.
